# How to prevent and address safeguarding concerns in global health research programmes: practice, process and positionality in marginalised spaces

**DOI:** 10.1136/bmjgh-2019-002253

**Published:** 2020-05-13

**Authors:** Bachera Aktar, Wafa Alam, Samiha Ali, Abdul Awal, Margaret Bayoh, Ivy Chumo, Yirah Contay, Abu Conteh, Laura Dean, Skye Dobson, Jerker Edstrom, Helen Elsey, Nadia Farnaz, Surekha Garimella, Linsay Gray, Jaideep Gupte, Kate Hawkins, Beth Hollihead, Kunhi Lakshmi Josyula, Caroline Kabaria, Robinson Karuga, Joseph Kimani, Alastair H Leyland, Dolf te Lintelo, Bintu Mansaray, Joseph MacCarthy, Hayley MacGregor, Blessing Mberu, Nelly Muturi, Linet Okoth, Lilian Otiso, Kim Ozano, Ateeb Parray, Penny Phillips-Howard, Vinodkumar Rao, Sabina Rashid, Joanna Raven, Francis Refell, Samuel Saidu, Shafinaz Sobhan, Prasanna Subramanya Saligram, Samira Sesay, Sally Theobald, Rachel Tolhurst, Phil Tubb, Linda Waldman, Jane Wariutu, Lana Whittaker, Haja Wurie

**Affiliations:** 1 BRAC University James P Grant School of Public Health, Dhaka, Dhaka District, Bangladesh; 2 Federation of Urban and Rural Poor, Freetown, Sierra Leone; 3 African Population and Health Research Center, Nairobi, Kenya; 4 Sierra Leone Urban Research Centre, Njala University, Freetown, Sierra Leone; 5 Liverpool School of Tropical Medicine, Liverpool, Liverpool, UK; 6 Slum Dwellers International, Cape Town, South Africa; 7 Institute of Development Studies, Brighton, Brighton and Hove, UK; 8 Health Sciences, University of York, York, UK; 9 The George Institute for Global Health, New Delhi, India; 10 University of Glasgow, Glasgow, Glasgow, UK; 11 Pamoja Communications, Brighton and Hove, United Kingdom; 12 LVCT, Nairobi, Kenya; 13 Slum and Shack Dwellers International Kenya, Nairobi, Kenya; 14 College of Medicine and Allied Health Sciences, University of Sierra Leone, Freetown, Western Area, Sierra Leone; 15 Slum Dwellers International, Mumbai, India; 16 CODOHSAPA, Freetown, Sierra Leone

**Keywords:** health policy, health systems, health services research

## Abstract

Safeguarding is rapidly rising up the international development agenda, yet literature on safeguarding in related research is limited. This paper shares processes and practice relating to safeguarding within an international research consortium (the ARISE hub, known as ARISE). ARISE aims to enhance accountability and improve the health and well-being of marginalised people living and working in informal urban spaces in low-income and middle-income countries (Bangladesh, India, Kenya and Sierra Leone). Our manuscript is divided into three key sections. We start by discussing the importance of safeguarding in global health research and consider how thinking about vulnerability as a relational concept (shaped by unequal power relations and structural violence) can help locate fluid and context specific safeguarding risks within broader social systems. We then discuss the different steps undertaken in ARISE to develop a shared approach to safeguarding: sharing institutional guidelines and practice; facilitating a participatory process to agree a working definition of safeguarding and joint understandings of vulnerabilities, risks and mitigation strategies and share experiences; developing action plans for safeguarding. This is followed by reflection on our key learnings including how safeguarding, ethics and health and safety concerns overlap; the challenges of referral and support for safeguarding concerns within frequently underserved informal urban spaces; and the importance of reflective practice and critical thinking about power, judgement and positionality and the ownership of the global narrative surrounding safeguarding. We finish by situating our learning within debates on decolonising science and argue for the importance of an iterative, ongoing learning journey that is critical, reflective and inclusive of vulnerable people.

**Summary box**

- Safeguarding challenges in global health research are shaped by power relations (eg, gender, age) and context (eg, informal urban spaces) and include sexual abuse and exploitation, physical and psychological abuse, exploitation and neglect.
- The literature on safeguarding in global health research is very limited; documented participatory processes that capture the situated knowledge, experience, difficulties and practice of different actors is required across varied contexts and health issues.

- Safeguarding processes need to be committed to changing power relations through the use of approaches that build trust and are centred around the needs of survivors.

## Introduction

Following high-profile cases of abuse involving staff in international NGOs, safeguarding concerns have rapidly risen up the agenda for donors and organisations that are funding and providing services to vulnerable groups. This has led to a number of recent initiatives within international development programmes and research. In the UK, these include a debate in the House of Commons (31 July 2018)^1^ and a government-hosted Safeguarding Summit (18 October 2018).^2^ Most recently, the UK Collaborative on Development Research (UKCDR) commissioned a Safeguarding in International Development Research enquiry to explore safeguarding across research and development and to support the generation of potential guidance and guidelines for good practice.^3 4^ This practice paper responds to the call within the UKCDR report for stakeholders to share their own learning of safeguarding best-practices, and to reflect on the processes and practices within international research consortia. Safeguarding is a challenging term to define and varies across contexts. In some settings, a key concern may be the potential for power relations of an authority to overrule individual autonomy in the name of protection and vulnerability; in others, the key concern may be inadequate attention given to the harm caused by abuse. Of course, both extremes remain risks in all situations. UKCDR define safeguarding in international development research as preventing and addressing “*any sexual exploitation, abuse or harassment of research participants, communities and research staff, plus any broader forms of violence, exploitation and abuse… such as bullying, psychological abuse and physical violence*”.^3 4^ We draw on this definition throughout this article.

Much of the current discussion on safeguarding comes from the perspective of the humanitarian sector and direct service provision and implementation. Yet researchers working in global health also experience safeguarding challenges and research donors require assurance that safeguarding processes and policies are developed and implemented to protect participants and researchers. Partners (researchers and funders) in the recently funded United Kingdom Research and Innovation (UKRI) Global Challenge Research Fund (GCRF) research hubs (details about the hubs can be found at https://www.ukri.org/research/global-challenges-research-fund/interdisciplinary-research-hubs-to-address-intractable-challenges-faced-by-developing-countries/) have discussed safeguarding and the need to share experiences, processes and practices. Our aim in this paper is to share process and practice relating to safeguarding within the GCRF Accountability for Informal Urban Equity Hub—known as ARISE. ARISE is a new research consortium, aiming to enhance accountability and improve the health and well-being of marginalised people living and working in informal urban spaces in low-income and middle-income countries, with research (initially) in Bangladesh, India, Kenya and Sierra Leone. (The ARISE partnership is led by LSTM and includes (in alphabetical order) African Population and Health Research Centre (APHRC); College of Medicine and Allied Sciences (CoMAHS) Sierra Leone; Glasgow University; Institute of Development Studies (IDS); James P Grant School of Public Health, BRAC University; LVCT Health; Sierra Leone Urban Research Centre (SLURC), Slum/Shack Dwellers International (SDI); The George Institute (TGI), India; and York University.) The consortium brings together researchers, service providers and federations of slum dwellers from these countries and the UK, and will work with marginalised communities. We structure the paper in three sections. First, we consider why safeguarding is of critical concern for research consortia. Second, we describe the processes we have initiated to develop a shared approach to safeguarding. Finally, we reflect on our current learning and challenges, and pose questions for future policy and practice.

## Why is a focus on safeguarding in global health research and development essential?

Safeguarding challenges in global health research vary depending on a wide range of factors, including the context and focus of research and are not limited to sexual abuse and exploitation, but include physical and psychological abuse, exploitation and neglect. While children are understood as inherently vulnerable, the question of who may be considered as ‘vulnerable adults’ is highly dependent on context and to some extent on the focus and process of research. In biomedical research, ‘vulnerable adults’ are often defined as those who have a constrained ability to consent (such as people with cognitive disabilities) or those who are positioned as biologically vulnerable (such as pregnant women). However, understanding vulnerability as a relational concept widens its potential scope significantly. Here, vulnerability is not a property of an individual but a relationship between individuals and others in specific times and places.^5^ Vulnerability is most commonly created in relationships of unequal power. These may be institutional, that is, due to hierarchies such as employer–employee relationships, or control over resources by an individual by virtue of their institutional position. However, such institutional relationships are also located outside institutions within wider systems of power and axes of inequity, such as social class, gender, caste, sexuality, age, disability, ethnicity, affluence and citizenship. Vulnerabilities therefore occur in situations of interpersonal power imbalances that are shaped by these wider power structures. For example, the much publicised reports of sexual abuse refer to male international aid workers as perpetrators and civilian or refugee women as victims, although a range of gender constellations are possible.^6^ Feminist analyses have highlighted that sexual abuse usually involves differential power relations in which “international workers exercise power over vulnerable populations by virtue of their ability to control the distribution and allocation of essential resources (eg, food, water, shelter) for human survival. At the very site of this interaction abuse occurs; for example, when international workers exchange food for sexual services”.^7^

Like wider development activities, research processes inevitably interact with existing societal relations of power, and therefore exacerbate, challenge or subvert these, by creating new relationships or by reconfiguring existing ones. Such relationships include those between researcher and ‘researched’, between members of research teams (international and national and including co-researchers and non-researchers), and between researchers and wider communities where research is taking place. This is true of research processes based only within one country (eg, the UK) or across international contexts. However, such power relations have an ‘added’ dimension when the ‘researched’ population are frequently dependent on research or developmental organisations for the provision of services and or amenities. Since social power relations are depend on and reflect context in terms of time and place, safeguarding in both research and development concerns vary across contexts. For example, people living in fragile or humanitarian contexts, refugee settlements and urban marginalised neighbourhoods may experience additional ‘layers’ of vulnerability, due to exacerbated or emergent power relations in situations of insecurity and rapid change.^3^

The extent to which researchers have the skills and resources to build supportive relationships is a key consideration. For example, are there accessible organisations they can refer people to if a safeguarding concern arises (eg, in the case of sexual and gender-based violence)? Are researchers aware of these and are resources available to support referral? And how does such a referral impact within their ‘host’ community? Are vulnerable people who are referred confident that they will get the services/support needed in referral facilities? And what challenges will they face from their community? Developing processes that support researchers, research teams and their institutions becomes essential in enabling them to navigate complex and ever-changing power relations to ensure they are responsive to intersecting vulnerabilities that may present safeguarding concerns.

## What did we do? Developing a shared approach to safeguarding within ARISE

Recognising the critical importance of safeguarding in research practice, within ARISE, we collectively decided to follow four core steps that allowed us to learn from each other and to co-develop understandings and approaches to safeguarding that will continue to evolve.

### Step 1: Sharing institutional guidelines and practice from across the hub

Some of our ARISE institutions already had Safeguarding Guidelines or Policies in place. Others had policies related to child protection, prevention of sexual harassment, child labour and whistle-blowing policies which were not explicitly labelled as safeguarding but included some safeguarding concerns, for example, sexual assault, abuse and gender-based violence. We shared both country-specific and international policies and guidelines regarding safeguarding. Table 1 provides an overview of the legislation and policy available to all ARISE partner institutions, disaggregated by country, and by focus (eg, children, adults, sexual and gender-based violence, disability) in relation to safeguarding (this table is illustrative and does not contain all relevant policy).

**Table 1:** Legislation and policy available related to safeguarding across the ARISE hub countries

	Bangladesh	India	Kenya	Sierra Leone	UK
**International policies**					
UNICEF (1989) The UN Convention on the Rights of the Child (ratified by the General Assembly Resolution on 20 November 1989)					
United Nations (1979) Convention for the Elimination of all Forms of Discrimination Against Women (ratified by the General Assembly Resolution on 18 December 1979)					
United Nations (1993) The Declaration on the Elimination of Violence against Women. United Nations (ratified by the General Assembly Resolution on 19 December 1993)					
Inter-Agency Standing Committee (2016). Protection against Sexual Exploitation and Abuse (PSEA). Global Standard Operating Procedures. May 2016					
Keeping Children Safe (2014). Child safeguarding standards and how to implement them					
CHS Alliance, Group URD and the Sphere Project (2014) Core Humanitarian Standard on Quality and Accountability					
CHS Alliance (2017) PSEA implementation quick reference handbook					
**Regional policies**					
			The African Charter on the Welfare and Rights of the Child (ACWRC) of 2002		
**Subject area**	**National-level policy and/or legislation**				
**Children**	National Children Policy (2011)	Protection of Children from Sexual Offences (POSCO) Act (2012, amended 2019)	The Constitution of Kenya (2010)	The Constitution of Sierra Leone (1991) (Currently under review)	House of Commons International Development Committee (2018) Sexual exploitation and abuse in the aid sector. 8th report of the session 2017 to 19. HC 840. 23 July 2018. Published 31 July 2018
	Early Marriage Protection Act (2017) MoWCA	UN Convention on the Rights of the Child (1990)	The Children Act Kenya (2010)	The Child Rights Act Sierra Leone (2007)	Charity Commission (2014). Policy paper. Safeguarding Children and Young People. 14 July 2014
	Child Protection Policy (Bangladesh Shishu Adhikar Forum)	Revised Integrated Child Protection Scheme India (2014). Ministry of Women & Child Development Government of India	Framework for the National Child Protection System Kenya (2011) National Council for Children’s Services	National Child Welfare Policy (2013)	Children’s Act (1989) (legislation.gov.uk)
**Vulnerable groups**		Constitution of India (1950): provisions for vulnerable groups, including women, scheduled castes, scheduled tribes, persons with disabilities, children, persons living with HIV and the aged	The Persons with Disability Act Kenya 2012	The Persons with Disability Act Sierra Leone (2011)	UK Policy Governance Association (2006). Act of Parliament. Safeguarding Vulnerable Groups Act 2006 (chapter 47)
				The National HIV and AIDS Commission Act (2011)	Office of the Public Guardian (2017). Policy paper SD8: office of the Public Guardian safeguarding policy (web version) Updated 4 July 2017.
**Gender-based violence (GBV**)	National Action Plan to Prevent Violence Against Women and Children (2013 to 2015)	The Protection of Women from Domestic Violence Act (2005)	Protection against Domestic Violence Act (2015)	Domestic Violence Act (2007)	The Domestic Violence, Crime and Victims Act (2004) (legislation.gov.uk)
	Domestic Violence (Protection and Prevention) Rule (2013) MoWCA				
**Sexual exploitation, abuse and harassment (SEAH**)			Sexual Offences Act (2006)	The Sexual Offences Act 2012 Sierra Leone (currently under review)	UUK (2016) Changing the Culture: Report of the Universities UK Taskforce examining violence against women, harassment and hate crime affecting university students
(**International) Research ethics**	National Research Ethics Committee Bangladesh. Directorate General of Health Services. Government of the People’s Republic of Bangladesh https://dghs.gov.bd/		Research Ethics guidelines	The Ethics Review Board Guidelines on Conducting Research	AREC (2013) A Framework of Policies and Procedures for University Research Ethics Committees
	Bangladesh Medical Research Council. https://bmrcbd.org/				UUK (2019) The Concordat to Support Research Integrity
					David Orr *et al* (2019) Safeguarding in international development research: evidence review. A report commissioned by UKCDR
**Organisational policies**					
**Children and** **vulnerable adults**	BRAC Child Protection Policy	SPARC follows a strict anti-child labour policy and therefore does not employ underage children for any work, and this is legislated by law	Guidelines for Conducting Research on Adolescents		Liverpool School of Tropical Medicine (LSTM) Safeguarding Policy
	Child and Adult Safeguarding Policy (World Vision, 2018)				LSTM Protecting Children and Vulnerable Adults Policy
	Child Safeguarding in Project Based Practice Guidance (2016)				LSTM Code of Conduct
	Child Safeguarding Policy (Save the Children) (2019)				LSTM Safeguarding Policy (for External Collaborating Partners)
	Organizational Child Protection Policy and Code of Conduct (2017, GRAMBANGLA UNNOYON)				LSTM Safeguarding Students Policy
	BRAC University Code of Conduct				

Entries are intended to be illustrative rather than representative of all possible legislation, guidelines and policy.

### Step 2: A participatory process within the hub to agree a joint definition of safeguarding

**Figure 1:**
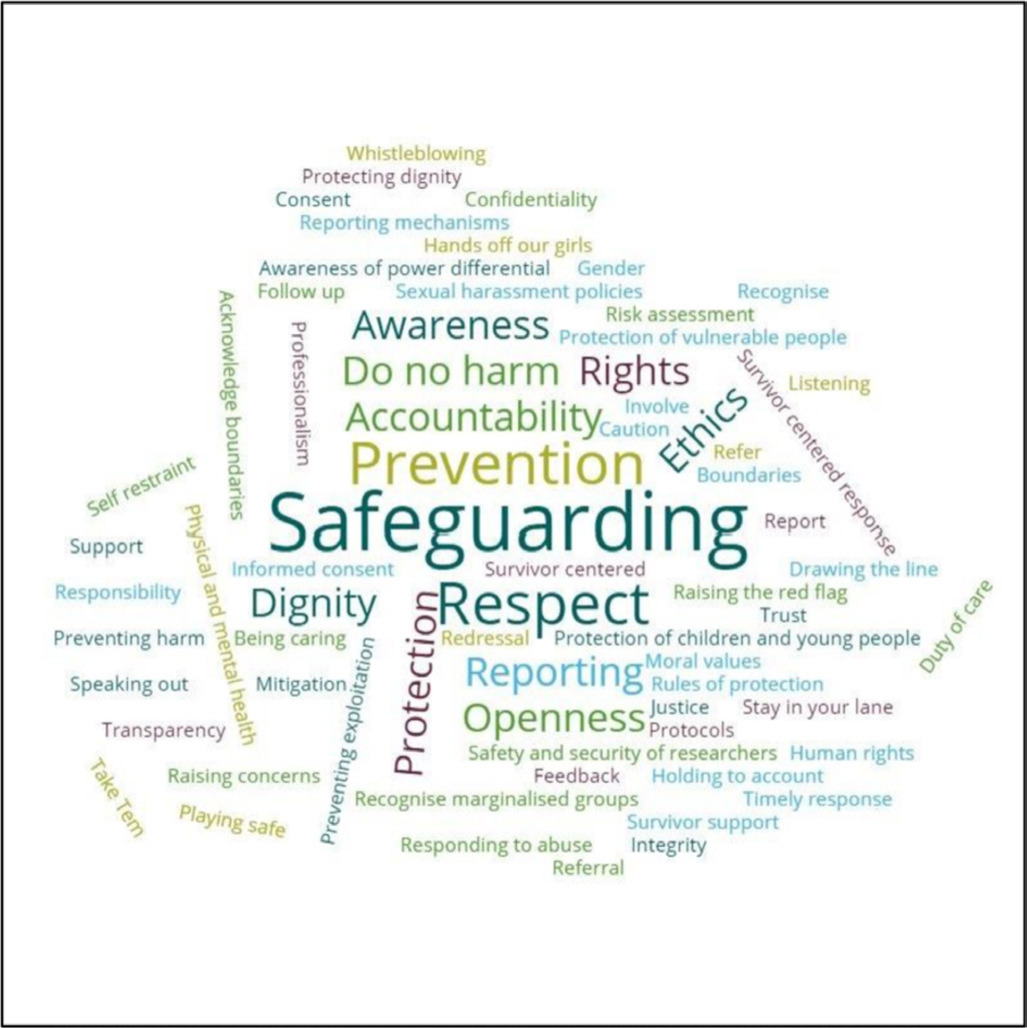
The ARISE collaboratively developed ‘word cloud’ to explore understandings and definitions of safeguarding.

During the ARISE hub inception meeting in Nairobi in February 2019, Liverpool School of Tropical Medicine’s (LSTM) lead safeguarding officer (PT) facilitated a face-to-face participatory learning exchange to discuss vulnerabilities and risks, identify practices and share experience across the partnership. This supported the development of shared ownership of safeguarding and preventing sexual exploitation, abuse and harassment across the hub. The exercises included a collective discussion about various terms and phrases that should be considered within safeguarding definitions. A safeguarding ‘word cloud’ was then developed (see figure 1) to visualise our shared understanding of safeguarding, and to use and reflect on this during the ARISE programme of work. The most frequently occurring word or phrases are shown in larger font.

Through the development of this word cloud and discussion, we then developed a working definition of safeguarding for ARISE as follows:

Safeguarding is a framework to protect children and vulnerable adults and prevent harm. Our research programmes will treat participants and their communities with dignity and respect and we will ensure systems are in place to empower the communities and our programme staff to openly speak out about abuse of power, including but not limited to sexual abuse, child abuse and exploitation and report and respond to safeguarding concerns

### Step 3: Developing, discussing and finalising the ARISE safeguarding vulnerabilities and risk assessment

We then worked in country teams to discuss safeguarding risks (noting that these are social and physical and shaped by existing vulnerabilities based on social power relations and the built environment) and feasible mitigation strategies. We adapted a safeguarding risk matrix developed at LSTM to create an ARISE Safeguarding Risk Mapping Tool (see online supplementary file 1) to facilitate discussion of potential risks faced by participants and researchers as a result of the study, risks unrelated to the study and any other potential risks. The exercises encouraged critical reflections from people of their previous experiences, what they could/should have done differently and the barriers to responding, such as a lack of available or accessible referral services. These were used as a basis for discussing how we could strengthen safeguarding processes throughout the lifetime of ARISE to ensure a lasting contribution in this area.

10.1136/bmjgh-2019-002253.supp1 Supplementary data

Following the inception meeting where this process was initiated, each ARISE partner from Kenya, Sierra Leone, Bangladesh and India completed a risk assessment, through team reflection and discussion. Risk assessments include information on (1) safeguarding risk identification and other risks, (2) safeguarding legislation and service provision and (3) developing an Action Plan. See table 2 for a summary of core risks and mitigation strategies identified across the consortium.

**Table 2:** Summary of key safeguarding concerns/risks identified across ARISE

LSTM safeguarding risk mapping tool						
LSTM department:	International Public Health	Programme title:	ARISE			
Summary:	An international research consortium aiming to enhance accountability and improve the health and well-being of marginalised people living and working in informal urban spaces					
Start date:	19 Feb	End date:	24 Feb	Countries:	Bangladesh, India, Kenya, Sierra Leone	
Principal investigator:	Professor Sally Theobald (LSTM)		Programme manager:	Beth Hollihead (LSTM)	Donor:	GCRF
Has LSTM signed up to a donor safeguarding policy or code of conduct under this grant?		Yes	Does the programme use volunteers? (if yes, detail role)			No
List all collaborating partners organisations working on this programme		Bangladesh: James P Grant School of Public Health/BRAC India: George Institute, Slum Dwellers International (SDI) Kenya: APHRC, LVCT Sierra Leone: COMAHS, SLURC UK: LSTM, Institute of Development Studies (IDS), Glasgow University, York University (SDI also work in Kenya and Sierra Leone)				
**Safeguarding risk identification**		Risks			How will the risks be mitigated/managed?	
1. Potential safeguarding/protection risks for beneficiaries that may occur within/as a result of undertaking the research?		Potential risk of SEAH to participants from people of trust such as researchers, co-researchers, security staff etc Potential risk of financial exploitation of participants from people of trust such as researchers, volunteers, partners, consultants, security staff etc Demands for accountability may make people vulnerable if powerholders interests are compromised Renewed trauma to participants by them reliving their experience by talking to you Lack of referral pathways leading to protection needs being unmet			Staff training Encouragement of reporting incidents/concerns Identification of appropriate organisations to refer to and appropriate referral pathways Strong institutional policies for child protection and anti-sexual harassment Sensitise staff on policies and signpost to them Sensitise communities and staff (on what to do and what not to do) Male/female pairs	
2. Potential safeguarding risks for staff, students, volunteers, contractors, consultants or visitors?		Potential risk of SEAH to researchers, volunteers, partners, consultants, security staff etc Risk of psychological harm from listening to trauma survivors Harassment of researchers, volunteers, partners, consultants, security staff when carrying out their work as part of this research programme Potential risk of burnout/distress of researchers researchers, co-researchers, security staff open to manipulation and corruption			Debrief, support and supervision available for the field research team Counselling services for the research team dealing with sensitive topics Boundary setting Male/female pairs Data collection and project activities conducted in groups/dyads, preferably mixed gender	
3. Safeguarding issues that could arise unrelated to the research activity?		Child abuse (eg, physical abuse, neglect etc) Sexual exploitation abuse or harassment (SEAH) unrelated to research Child, early or forced marriage (CEFM), gender-based violence (GBV) or intimate partner violence (IPV) Female genital mutilation (FGM) Eviction/homelessness Drug/alcohol/substance abuse/crime Violent crime Community tensions cultural norms, stigma against certain groups Religious or cultural practices Natural/sudden-onset disasters leading to safeguarding issues (homelessness, unaccompanied children etc)			Establish report and referral mechanism/procedure Orientate researchers on relevant national laws and policies in relation to protection of children and vulnerable adults	
4. Other risks identified (including moral and ethical risks of the research, health, safety and security risks)		Data protection and security of data Opportunity costs to participants of taking part in research Stigma of taking part in the research False hope on perceived benefits shapes participation in the study Unintended negative consequences because of participation in the study (ie, violence, social isolation, bullying etc) Perceived as being an agent for someone else, eg, city councils, which may lead to eviction Physical and psychological health risks to researchers and other staff, partners and volunteers from working in the community/within the political context Health, safety and security risks to researchers and other staff, partners and co-researchers while working in the community/within the political context Corruption/organised cartels Researchers/volunteers other staff and partners not being aware of cultural or religious norms while working in the area			Orientation for staff on research methods, ethics and cultural sensitivity Understand power dynamics of the community/study population before starting the research Inclusive and participatory methodology Adequate briefing and preparation for research team Provide safety guidelines and sensitise research team Inform local authorities about the research (ie, city corporation, police, NGOs etc) Support of federation networks is advantageous since the ground realities are mainly known beforehand and therefore the opportunity to orient all those involved mitigates the distress and there are lesser situational unknowns. Much more local support is available if the ground situation gets tenuous Engage clearly with gatekeepers, chiefs and others	

There are many similarities in vulnerabilities experienced by urban marginalised people across the participating countries, including endemic violence and abuse, harassment from official agencies, and limited availability of and access to responsive services (such as health and social services and police). Furthermore, insecurity, powerlessness, lack of information and recognition of rights to report abuse, and access to limited services are enmeshed in a complex web of power relations and marginalisation related to poverty, class and caste, tenancy rights, age and generation, (dis)ability, and patriarchal norms of gender and sexuality. These constitute a context of structural violence that poses particular challenges for operationalising safeguarding processes.

### Step 4: Ongoing review of action plans through a process of implementation, learning and reflection

Our next steps are to implement actions. We have identified safeguarding leads in each partner institution to spearhead this process and facilitate context specific ownership and learning. As a consortium, we will encourage reflective practice and the sharing of dilemmas and good practice through online knowledge sharing platforms (eg, webinars, blogs and discussion forums) and learning exchanges. We are currently collaboratively developing an overarching, cross-organisational safeguarding policy and procedures to describe the cohesive approach that we are adopting across the consortium, and which defines a shared reporting mechanism for ARISE. This will keep safeguarding at the forefront during annual consortium meetings and regular meetings of executive and advisory groups.

## What did we learn?

### Who owns the narrative? Power, positionality and reflective practice

The impetus to set up systems and processes for safeguarding has largely been driven by UK-based donors, responding to concerns about sexual exploitation of beneficiaries by staff working for international non-governmental organisations. Such exploitation may be widespread, but it has been made visible by a small number of high-profile public cases. We need to approach safeguarding with a critical lens, given calls to decolonise science,^8^ and to problematise the analysis, institutions and processes “that animate the global health space”.^9^ We also need to situate our discussions on safeguarding within our agreed ARISE values of equitable practice, transparency and accountability, continuous co-learning and ethical practice. Orr and colleagues,^3^ within their UKCDR-commissioned report, document tensions between establishing guidelines and policies to meet the much-needed safeguarding demands of international donors (shaped by power and rooted in colonial legacies), while also establishing equitable global research partnerships. In addition to global economic and gendered power relations underpinning abuse by international development workers, class, gender, caste and other hierarchies within low-income and middle-income countries also enable abuse of power and lack of accountability and transparency in organisations, with survivors often facing backlash or injustice. This resonated across our ARISE partnership, reflecting on our own positionalities. Where the onus for action on safeguarding was coming from was an important part of our discussions. Hence, the importance of embedding a participatory process to ensure the agenda and processes we develop speak to the realities, concerns and embedded knowledge of all partners. However, we also must recognise that those of us who have engaged within these participatory processes (key researchers at all ARISE partner institutions) are also in relative positions of power within our own contexts. Considering how to seek the views of those who are more marginalised in informing both our understandings of safeguarding and the development and implementation of our safeguarding action plans will be critical as we move forward. For example, within our discussions around safeguarding to date, the views and experiences of people with disabilities has been largely absent. We have taken steps in Sierra Leone to engage people with disabilities as co-researchers and, as a key part of the engagement process, sought to understand their views and experiences regarding specific safeguarding issues. However, this is just a small step in ensuring that marginalised groups, such as people with disability, are continuously included in navigating and shaping safeguarding discourse within global health.

Another key finding in the Orr *et al*^3^ report is that “safeguarding is an unfamiliar term to many and barely featured in the international literature”.^3^ Rather, other terms such as exploitation and harm that share similar values to safeguarding are more widely discussed. Thus, the wealth of experience in relation to safeguarding among researchers across contexts, particularly those working in low-income and middle-income countries, is challenging to identify when only considered if it has been previously categorised as work directly related to safeguarding.^3^ The participatory process we went through as a consortium supported this finding and evidenced that the UK partners, who need to report on this, often have the least contextual and embedded knowledge of daily realities in research sites. This recognition is essential to ensure locally owned safeguarding agendas. While many processes and understandings evident on the ground may not be documented in literature of ‘high academic value’ or ‘international stature’, there is significant commitment to safeguarding principles, tacit and local knowledge and best practice in relation to safeguarding within many low-income and middle-income partner institutions. The challenge now is for all partners to listen, learn and respond to our varied expertise and knowledge to create a jointly owned agenda and process.

### Who has safeguarding expertise? What constitutes a safeguarding issue and who decides?

The embedded knowledge of ARISE partners based in Bangladesh, Kenya, India and Sierra Leone, on the range of daily challenges facing marginalised people living and working in informal urban spaces and the potential safeguarding vulnerabilities for researchers and participants, was clear from the discussions. This includes lived knowledge and experience of marginalised people living in informal spaces through SDI in India, Sierra Leone and Kenya; and expertise in identifying and responding to sexual and gender-based violence cases, including child sexual abuse in Kenya and Sierra Leone and Bangladesh (such as the BRAC Gender Justice and Diversity Programme). Each partner brought their own positionalities and perspectives. The UK partners, in contrast, often realised how little they knew about the realities of daily life, vulnerabilities and complexities of response in these specific contexts.

In our discussions and in the safeguarding assessments, there was active debate around what constitutes a safeguarding concern and how this may differ from health and safety and ethical concerns. There are clearly some overlaps. The safeguarding risk assessment process identified several safeguarding concerns that were shared across the hub (table 2). Other common emerging concerns related to participants and research safety but may not be conventionally classified as safeguarding. For example, does a research team member’s risk of experiencing crime or violence while conducting research in urban informal spaces represent a safety and security issue or a safeguarding issue? If a participant is distressed by a research encounter, for example through biased or judgemental questioning or through revealing experiences of violence or harm, is this a safeguarding issue or an ethical issue? On discussion and reflection, we felt it is positive that the assessment of safeguarding risks triggered a wide-ranging consideration of broader concerns. This led the LSTM team to structure the risk assessments into ‘safeguarding’ and ‘other’ while acknowledging that some concerns could still fall into multiple categories. We are conscious of the need to ensure that concerns feed into ethics protocols and ongoing good ethical research practice as well as health and safety policy and practice, and this was included as a key step in action planning.

At this point, however, we need to consider our own positions of power in making the decisions on ‘what constitutes safeguarding’ and ‘what does not’. By its very nature, safeguarding is designed to protect the most vulnerable from those in a position of power; should it not then be for the most vulnerable to decide what constitutes safeguarding? Or is it the ethical responsibility of those in positions of relative power to make these decisions? This is challenging if we use a relational and temporal concept of vulnerability. For example, all marginalised people living and working in urban informal spaces may be considered as vulnerable, but within relationships and public spaces young women and men may experience these vulnerabilities differently. Young women may be particularly vulnerable to sexual and gender-based violence; however, patriarchal social norms may prevent these young women and the wider community perceiving and raising their vulnerability as a safeguarding issue. Members of research teams may potentially be both perpetrators and victims/survivors of safeguarding violations (including within research teams and between researchers and participants). As we move forward in the safeguarding process, we will continue to create opportunities for urban marginalised people to participate in review processes as we begin to implement our procedures. Throughout this process, we will be guided by our core shared values of striving towards equity by challenging existing inequities shaped by gender, class, caste, sexuality and disability, while mindful of tensions between culturally located and universalist values.

### What safeguarding practices are feasible? Implementation of legislation and service provider availability in urban informal spaces

Safeguarding is a challenge for research teams who are not service providers in terms of responding effectively to issues identified. Within the ARISE consortium, only one partner, LVCT Health, is a service provider able to build capacity of health providers to screen for and provide medical, psychological and legal response or referral for victims of gender-based violence in community, public and private facility settings. LVCT Health pioneered the first guidelines and training manual for post-rape care services in Kenya with the government in 2004, with many subsequent revisions. Currently, 20 000 survivors of sexual violence have been offered post-rape care services by LVCT Health and 4000 professionals in the justice system (police officers, healthcare providers, lawyers and magistrates) have been trained on how to address cases of sexual violence.^10^

Discussions and assessments revealed a wide range of legislation in place (table 1). However, the capacity and willingness of the state and other governance actors to implement such legislation in urban informal spaces is, to varying extents, limited. Perpetrators of some forms of safeguarding violations may be state actors, including the police, or embedded respected community members. For example, SDI colleagues in Kenya found that involving young people living in urban informal spaces in research processes increased their vulnerability to police harassment because the police assumed that the electronic devices they used for collecting data had been stolen. State designation of urban areas as ‘informal’ or even ‘illegitimate’ often means that even services available in the ‘formal’ city are not provided. In our low-income and middle-income contexts, services such as child protection, social support and legal redress for sexual and gender-based violence are generally limited and often not geographically or financially accessible for the majority of urban dwellers. Stigma may compound these issues for some further marginalised people such as sex workers or LGBTI people.

As employers, research institutions have a duty of care to ensure that appropriate services are provided to their staff if they experience a safeguarding violation, or to participants in research if harm occurs as part of the research. However, it is beyond the capacity of most ARISE partner institutions to ensure accessible service provision for all vulnerable people experiencing violence and abuse that is visible to the research team. Our research programme has the responsibility to facilitate processes at a collective level to challenge structural violence, including lack of service provision, and to promote an equitable social environment in which violence and abuse is less likely to occur and in which vulnerable individuals know their rights and feel able to disclose abuses. We will also endeavour to identify and support existing ‘informal’ processes for protecting vulnerable children and adults from abuse developed by people with shared values in these contexts, where resilience and innovation are core features of daily survival for many.

## Conclusion

The safeguarding agenda is a relatively new framing, particularly in the global health research arena, of issues in research and development that have long concerned feminists and ethicists. Discussing and developing processes to promote safeguarding within large-scale research networks in global health is an important and positive step, and requires partnerships, participatory processes, reflexivity and the building of trust. Honest discussions and critical self-reflection are needed on the strategies being used to promote safeguarding, to monitor how well these are working and to identify ways that they may be improved by learning from each other. The implementation of safeguarding agendas needs to be guided by the lived realities of the most marginalised, not by researchers in the ‘global north’ to teach or impart wisdom. Genuine ownership and engagement are needed by all partners to create, implement and adjust practices in challenging contexts. We aim to share our experiences with the wider research community. Future work that explores the similarities and differences between the values and principles that underpin safeguarding and ethical practice is of critical importance. This will ensure that both discourses learn from each other and are given equal importance in the promotion of research integrity and that good practice in one area does not substitute that in another. As with research ethics, safeguarding is not merely a procedural check box process but an iterative, ongoing learning journey that is critical, reflective and inclusive of vulnerable people. Ultimately, safeguarding processes need to be situated in a critical understanding of power relations, committed to changing them and promoting equity.
