# Supplementary material for: How to prevent and address safeguarding concerns in global health research programmes: practice, process and positionality in marginalised spaces
Source: BMJ Glob Health. 2020 May 13;5(5):e002253. doi: 10.1136/bmjgh-2019-002253 (PMC7228499; doi:10.1136/bmjgh-2019-002253)
Supplement: Supplementary data [file bmjgh-2019-002253supp001.pdf]

| LSTM Safeguarding Risk Mapping Tool (adapted for use by the ARISE Programme) | | |
|---|---|---|
| **Safeguarding Risk Identification** | Risks | How will the risks be mitigated/managed? |
| 1. Potential safeguarding/protection risks for participants that may occur within/as a result of undertaking the research? | | |
| 2. Potential safeguarding risks for staff, students, volunteers, contractors, consultants or visitors? | | |
| 3. Safeguarding issues that could arise unrelated to the research activity? | | |
| 4. Other risks identified (including moral & ethical risks of the research, health, safety & security risks) | | |

LSTM Safeguarding risk mapping tool. Developed by P Tubb, LSTM.

| **Additional Information** | |
|---|---|
| What international and national legislation and/or guidance documents are available in relation to Safeguarding/Protection of children +/or vulnerable adults in the country you are working in? Please detail | What services are available locally as part of victim response for child / vulnerable adult protection? Please detail (e.g child protection, GBV services, HIV services) |
| | |
| | |
| | |
| | |
| | |
| | |

| **Action Plan. (What additional action (if any) do you now need to take to mitigate the risks identified)?** |
|---|
| |
| |
| |
| |
| |
| |

LSTM Safeguarding risk mapping tool. Developed by P Tubb, LSTM.
